# Psychiatric Correlates of Snuff and Chewing Tobacco Use

**DOI:** 10.1371/journal.pone.0113196

**Published:** 2014-12-23

**Authors:** Qiang Fu, Michael G. Vaughn, Li-Tzy Wu, Andrew C. Heath

**Affiliations:** 1 Department of Biostatistics, College for Public Health and Social Justice, Saint Louis University, St. Louis, Missouri, United States of America; 2 School of Social Work, College for Public Health and Social Justice, Saint Louis University, St. Louis, Missouri, United States of America; 3 Department of Psychiatry and Behavioral Science, Duke University Medical Center, Durham, North Carolina, United States of America; 4 Department of Psychiatry, School of Medicine, Washington University in St. Louis, St Louis, Missouri, United States of America; University of Southern California, United States of America

## Abstract

Compared to the association between cigarette smoking and psychiatric disorders, relatively little is known about the relationship between smokeless tobacco use and psychiatric disorders. To identify the psychiatric correlates of smokeless tobacco use, the analysis used a national representative sample from the National Epidemiologic Survey on Alcohol and Related Conditions (NESARC) wave 1. Smokeless tobacco use was classified as exclusive snuff use, exclusive chewing tobacco, and dual use of both snuff and chewing tobacco at some time in the smokeless tobacco user's life. Lifetime psychiatric disorders were obtained via structured diagnostic interviews. The results show that the prevalence of lifetime exclusive snuff use, exclusive chewing tobacco, and dual use of both snuff and chewing tobacco was 2.16%, 2.52%, and 2.79%, respectively. After controlling for sociodemographic variables and cigarette smoking, the odds of exclusive chewing tobacco in persons with panic disorder and specific phobia were 1.53 and 1.41 times the odds in persons without those disorders, respectively. The odds of exclusive snuff use, exclusive chewing tobacco, and dual use of both products for individuals with alcohol use disorder were 1.97, 2.01, and 2.99 times the odds for those without alcohol use disorder, respectively. Respondents with cannabis use disorder were 1.44 times more likely to use snuff exclusively than those without cannabis use disorder. Respondents with inhalant/solvent use disorder were associated with 3.33 times the odds of exclusive chewing tobacco. In conclusion, this study highlights the specific links of anxiety disorder, alcohol, cannabis, and inhalant/solvent use disorders with different types of smokeless tobacco use.

## Introduction

The use of smokeless tobacco is a significant yet understudied public health problem. According to the national health surveys, smokeless tobacco use among U.S. adults ranged from 0.8% to 9.2% [1], [2]. Smokeless tobacco products are a major source of carcinogenic nitrosamines and associated with increased risk for oral, esophageal, and pancreatic cancer [3]–[5]. The International Agency for Research on Cancer has classified smokeless tobacco as a human carcinogen [6]. In addition, smokeless tobacco use increases myocardial infarction and stroke [7]. In North America and parts of Europe, the most commonly used smokeless tobacco products include snuff (dry or moist) or (and?) chewing tobacco. Moist snuff is the most popular smokeless tobacco product used in the United States [8].

Smokeless tobacco is most commonly used by younger Caucasian men, some Native American and Alaska Native tribes, and residents of rural areas [8]. Smokeless tobacco use has been linked to elevated risk for alcohol, cigarette, and marijuana use along with depression in the National Institute on Drug Abuse National Household Survey [9]. The findings on the association between smokeless tobacco use and psychiatric disorders are mixed in the current literature. Previous studies have indicated that past-year smokeless tobacco users who met lifetime DSM-IV diagnostic criteria for nicotine dependence were associated with increased risk for specific phobia after controlling for demographic and psychiatric covariates as well as quantity of cigarettes smoked [10]. In contrast, past-year smokeless tobacco users without nicotine dependence were not associated with any psychiatric disorders [10]. These results were obtained based on the smokeless tobacco users who had used any smokeless tobacco products in the past 12 months prior to the interview and did not show whether both snuff and chewing tobacco were associated with specific phobia. Another report found that there was no association between exclusive smokeless tobacco use and cannabis use disorder, but the dual use of smoked and smokeless tobacco was (were?) associated with greater odds of cannabis use disorder [11].

Most previous studies did not measure psychiatric disorders [3]–[5], or restricted study inclusion to past year smokeless tobacco use rather than lifetime use [10], [11]. Thus, some lifetime smokeless tobacco users were misclassified as non-users, potentially missing important relations. Previous studies also did not differentiate any specific form of smokeless tobacco when relations of smokeless tobacco use with psychiatric disorders were examined [10], [11]. As a result, it is unknown whether psychiatric comorbidities are similar in solely snuff users, solely chewing tobacco users, or dual users. It is also unknown whether findings from past-year smokeless tobacco users can be generalized to lifetime users.

To address these gaps in smokeless tobacco research, the present study utilizes a large, nationally representative sample to clarify some ongoing issues in the study of lifetime smokeless tobacco use and lifetime psychiatric disorders. This study examined differences between lifetime smokeless tobacco users and non-users of smokeless tobacco in relation to psychiatric disorders. Moreover, this study delineated exclusive snuff use or exclusive chewing tobacco from dual use with respect to psychiatric disorders.

## Materials and Methods

### Sample

Data was derived from the National Epidemiologic Survey of Alcohol and Related Conditions (NESARC) wave 1 sponsored by the National Institute on Alcohol Abuse and Alcoholism (NIAAA) and conducted by the U.S. Bureau of the Census in 2001 and 2002. A sample of 43,093 non-institutionalized civilians 18 years and older was selected from the U.S. population. The person-to-person interview survey oversampled young adults, Hispanics, and African-Americans. The overall response rate was 81%. The sample was weighted and adjusted to reflect the U.S. population from the 2000 Decennial Census with respect to age, sex, race and ethnicity to account for non-response and selection probability. Details of the sampling frame and weighting method are published elsewhere [12]–[15]. All respondents provided a written informed consent to participate in the NESARC. The research protocol and informed consent procedures were reviewed and approved by the U.S. Bureau of the Census and the U.S. Office of Management and Budget.

### Measures

Psychiatric diagnoses were made using the Alcohol Use Disorder and Associated Disabilities Interview Schedule-IV (AUDADIS-IV), a computer- assisted interview software developed by the NIAAA,according to the Diagnostic and Statistical Manual of Mental Disorders IV (DSM-IV) [16]. Experienced professional interviewers from the U.S. Bureau of the Census were trained under the direction of NIAAA. A subsample was randomly selected and re-interviewed with one to three complete sections for the purpose of quality control and rest-retest reliability analysis [17]. These results indicated that the AUDADIS_IV possessed good reliability and validity [18]–[22].

#### Smokeless tobacco use

Lifetime smokeless tobacco users were defined if they answered affirmatively to the question in the NESARC, “In your entire life, have you ever used snuff, such as Skoal, Skoal Bandit or Copenhagen at least 20 times?” or “In your entire life, have you ever used chewing tobacco, such as Redman, Levi Garrett or Beechnut at least 20 times?”. Lifetime cigarette users were defined if they answered affirmatively to the question, “In your entire life, have you ever smoked at least 100 cigarettes?” The quantity of cigarettes was obtained according to the question, “Thinking back over the entire period when you were smoking every day, about how many cigarettes did you usually smoke in a single day?”

#### Sociodemographic covariates

Sociodemographic variables included age, sex, race/ethnicity, marital status, household income, educational attainment, metropolitan area, regions, and birth in the United States. Age was divided into the categories 18–34, 35–49, 50–64, and 65+. Race/ethnicity was classified into White, African-American, Hispanic, and American Indian/Alaska Native/Asian/Native Hawaiian/Pacific Island. Marital status was classified as never married, married, and widowed/separated/divorced. Household income was categorized as <$20,000, $20,000–$34,999, $35,000–$69,999, and ≥$70,000. Educational attainment was classified as less than high school, high school graduates, and college or higher education.Psychiatric diagnosis.

Lifetime DSM-IV psychiatric diagnoses included major depression, dysthymia, mania or hypomania, panic disorder, social phobia, specific phobia, general anxiety disorder, alcohol use disorder, nicotine dependence, cannabis use disorder, cocaine use disorder, sedatives use disorder, hallucinogens use disorder, opiates use disorder, tranquilizers use disorder, inhalants use disorder, antisocial personality disorder, avoidant personality disorder, dependent personality disorder, histrionic personality disorder, paranoid personality disorder, schizoid personality disorder, and obsessive-compulsive personality disorder. The test-retest reliability has been documented for mood and anxiety disorder diagnoses and symptom items (kappas 0.42–0.64) [17] for the alcohol dependence diagnosis (kappas 0.7–0.84) [17]–[20] and nicotine dependence diagnosis (kappa  = 0.63) [12], [14] [1], [2] respectively. Good validity of psychiatric diagnoses has also been documented elsewhere [21], [22].

### Statistical Analysis

All analyses were conducted using SUDAAN [23]. This software implements a Taylor series linearization to adjust standard errors of estimates for complex survey sampling design effects including clustered data. The dependent variable of smokeless tobacco use was recoded as exclusive snuff use, exclusive chewing tobacco, use of both snuff and chewing tobacco, and non-tobacco use (reference group). Based on the number of cigarettes smoked per day, an ordinal variable was recoded as 0, 1–5, 6–15, 16–29, 30+. Weighted percentages were used to report the prevalence of sociodemographic characteristics and psychiatric disorders by smokeless tobacco use status. Since a chi-square test is not very informative for contingency tables beyond 2×2 dimensions, we conducted a significance test using 95% confidence interval (CI) of prevalence. 95% CIs of two prevalence estimates not overlapping indicates *p*<0.05. We used multinomial logistic regression to detect associations between lifetime smokeless tobacco use and psychiatric disorders while controlling for sociodemographic covariates and quantity of cigarettes smoked. Adjusted odds ratios (OR) and 95% CI were reported to quantify the associations.

## Results

Table 1 displays the sociodemographic characteristics of respondents stratified by a history of smokeless tobacco use at some point in their life. Smokeless tobacco use was significantly more common in Caucasians, men, people aged 18 to 34 years old, those born in the United States, and residents of suburban and rural areas. Smokeless tobacco use was less common among African Americans and Hispanics. Exclusive chewing tobacco and use of both snuff and chewing tobacco were less likely to report in persons aged 50 years or older compared to non-smokeless tobacco use. Exclusive chewing tobacco was more common in people who were married, but less common in those who were never married compared to non-smokeless tobacco use. Those who were divorced, separated, widowed, or came from families with household incomes less than $20,000 were less likely to use only snuff or both snuff and chewing tobacco. There was no difference in prevalence between smokeless tobacco use and non-smokeless tobacco use among people with household incomes greater than $70,000. Persons who did not complete their high school education were more likely to chew tobacco only. High school graduates were more likely to use any smokeless tobacco products, but individuals with college or higher education were less likely to use both snuff and chewing tobacco or only chew tobacco.

**Table 1:** Sociodemographic characteristics of respondents stratified by non-smokeless tobacco use, exclusive snuff use, exclusive chewing tobacco, and dual use of snuff and chewing tobacco.

	Non-smokeless tobacco use(n = 23,442)	Snuff(n = 716)	Chewing tobacco(n = 901)	Snuff and chewing tobacco(n = 931)
	% (95%CI)	% (95%CI)	% (95%CI)	% (95%CI)
Sex				
Male	44.13 (43.47, 44.80)	**91.16 (88.76–93.09)**	**94.39 (92.67–95.72)**	**95.89 (94.30–97.05)**
Female	55.87 (55.20, 56.53)	**8.84 (6.91–11.24)**	**5.61 (4.28–7.33)**	**4.11 (2.95–5.70)**
Age				
18–34	31.08 (30.18, 32.00)	**47.98 (43.41–52.59)**	**23.12 (19.98–26.60)**	**40.46 (36.75–44.29)**
35–49	30.93 (30.30, 31.56)	33.36 (29.36–37.61)	30.43 (26.79–34.33)	**36.20 (32.53–40.03)**
50–64	21.41 (20.88, 21.95)	**12.15 (9.78–15.01)**	24.50 (21.22–28.10)	**14.67 (12.24–17.49)**
65+	16.58 (15.88, 17.30)	**6.51 (5.03–8.39)**	**21.95 (19.14–25.05)**	**8.68 (6.89–10.87)**
Race				
Black	11.53 (10.26, 12.93)	**3.26 (2.33–4.54)**	**8.17 (6.53–10.19)**	**4.02 (3.05–5.27)**
Hispanic	12.13 (9.79, 14.94)	**4.33 (2.89–6.50)**	**3.61 (2.19–5.90)**	**4.11 (2.73–6.14)**
Indian/Alaska/Asian/Hawaiian-Pacific	6.65 (5.65, 7.81)	**4.36 (2.89–6.53)**	**4.45 (3.17–6.21)**	4.85 (3.35–6.97)
White	69.69 (66.32, 72.86)	**88.05 (85.33–90.33)**	**83.77 (80.84–86.33)**	**87.02 (84.27–89.36)**
Income				
<$19,999	23.88 (22.92, 24.87)	**16.95 (13.96–20.43)**	23.37 (19.78–27.40)	**19.26 (16.54–22.29)**
$20,000–$34,999	20.00 (19.33, 20.68)	21.53 (18.16–25.32)	20.55 (17.53–24.32)	21.84 (19.11–24.84)
$35,999–$69,999	31.82 (31.17, 34.47)	**38.64 (34.53–42.92)**	35.49 (31.54–39.64)	35.42 (31.97–39.02)
>$69,999	24.30 (22.90, 25.76)	22.88 (19.38–26.81)	20.59 (17.20–24.45)	22.88 (19.38–26.81)
Marital Status				
Never married	20.84 (19.89, 21.83)	23.43 (19.50–27.88)	**14.36 (11.50–17.79)**	20.50 (17.49–23.87)
Separated/divorced/widowed	17.70 (17.21, 18.20)	**14.06 (11.63–16.91)**	15.85 (13.58–18.41)	**13.90 (11.55–16.64)**
Married/Cohabitating	61.46 (60.51, 62.40)	62.51 (57.97–66.84)	**69.79 (66.19–73.16)**	65.61 (61.72–69.29)
Education				
<High school	15.42 (14.43, 16.47)	15.62 (12.74–19.01)	**22.11 (19.04–25.51)**	16.72 (14.02–19.82)
High school graduates	28.87 (27.77, 30.00)	**33.99 (29.74–38.51)**	**34.45 (31.00–38.08)**	**33.95 (30.33–37.77)**
College or higher	55.71 (54.41, 56.99)	50.39 (46.07–54.70)	**43.44 (39.66–47.31)**	**49.33 (45.20–53.46)**
Urbanicity				
Central city	30.19 (25.88, 34.89)	**19.21 (15.55–23.49)**	**20.87 (17.51–24.67)**	**20.93 (17.59–24.71)**
Suburban or rural	69.81 (65.11, 74.12)	**80.79 (76.51–84.45)**	**79.31 (75.33–82.49)**	**79.07 (75.29–82.41)**
Regions				
Northeast	20.14 (13.99, 28.11)	15.81 (10.49–23.13)	13.70 (9.38–19.60)	11.24 (7.93–15.69)
Midwest	23.02 (17.09, 30.25)	26.00 (20.76–32.02)	22.45 (17.47–28.35)	26.78 (21.81–32.40)
South	34.60 (28.30, 41.50)	36.60 (30.30–43.40)	47.23 (40.84–53.72)	42.99 (36.91–49.30)
West	22.24 (15.84, 30.30)	21.59 (16.61–27.58)	16.62 (11.94–22.66)	18.99 (14.03–25.19)
Born in US				
Yes	84.40 (81.05, 87.25)	**97.98 (95.18–99.17)**	**97.16 (95.33–98.28)**	**98.01 (96.87–98.86)**
No	15.60 (12.75, 18.95)	**2.02 (0.83–4.82)**	**2.84 (1.72–4.67)**	**1.90 (1.14–3.13)**
Cigarettes Smoked				
Yes	41.74 (40.43.03)	**70.12 (66.24, 73.72)**	**75.17 (71.66, 78.37)**	**74.37 (71.05, 77.44)**
No	58.26 (56.97, 59.54)	29.88 (26.28, 33.76)	24.83 (21.63, 28.34)	25.63 (22.56, 28.95)

Note: Boldface percentages are significantly different from those in the non-smokeless tobacco use group (p<0.05).

Table 2 shows the lifetime prevalence of psychiatric disorders and smoking among respondents divided by their history of smokeless tobacco use at some point in their life. Mania/hypomania was more likely to be associated with exclusive snuff use and use of both snuff and chewing tobacco. Obsessive-compulsive, paranoid, Schizoid, and histrionic personality disorders were more likely to be associated with use of both snuff and chewing tobacco. Antisocial personality disorder, and substance user disorders including alcohol, cannabis, amphetamine, opiates, sedatives, cocaine, and hallucinogens were significantly associated with all types of smokeless tobacco use compared to non-smokeless tobacco use. Tranquilizer, heroin and inhalant use disorders were significantly associated with exclusive chewing tobacco and dual use of snuff and chewing tobacco. Cigarette smoking was more likely to be associated with any types of smokeless tobacco use.

**Table 2:** Prevalence of lifetime psychiatric disorders and cigarette smoking among respondents stratified by lifetime use of smokeless tobacco.

	Non-smokeless tobacco use(n = 23,442)	Snuff(n = 716)	Chewing tobacco(n = 901)	Snuff and chewing tobacco(n = 931)
	% (95%CI)	% (95%CI)	% (95%CI)	% (95%CI)
Mood disorders				
Major depression	16.73 (15.93, 17.50)	16.74 (13.76–20.22)	13.48 (11.25–16.07)	18.80 (16.04–21.92)
Dysthymia	4.31 (4.02, 4.62)	4.00 (2.37–6.68)	4.86 (3.62–6.50)	5.36 (3.85–7.42)
Mania/hypomania	5.46 (5.12, 5.83)	**8.85 (6.44–12.06)**	7.31 (5.55–9.57)	**9.25 (7.45–11.42)**
Anxiety disorders				
Panic disorder	4.05 (3.78, 4.33)	3.33 (1.96–5.60)	5.35 (3.74–7.61)	3.14 (1.97–4.98)
Social phobia	4.96 (4.57, 5.38)	6.74 (4.66–9.65)	6.00 (4.22–8.47)	4.87 (3.46–6.81)
Specific phobia	9.48 (8.88, 10.12)	9.38 (6.96–12.54)	10.68 (8.44–13.44)	8.52 (6.49–11.11)
General Anxiety Disorder	4.20 (3.86, 4.56)	3.84 (2.55–5.74)	3.20 (2.21–4.59)	4.77 (2.99–7.54)
Personality Disorder				
Avoidant	2.32 (2.11, 2.56)	3.08 (1.78–5.26)	2.58 (1.53–4.31)	3.36 (1.94–5.77)
Dependent	0.48 (0.40, 0.58)	0.45 (0.12–1.76)	0.49 (0.17–1.41)	1.10 (0.42–2.86)
Obsessive-Compulsive	7.70 (7.26, 8.18)	10.37 (7.70–13.83)	9.90 (7.84–12.44)	**12.62 (10.23–15.47)**
Paranoid	4.32 (4.02, 4.64)	6.32 (4.29–9.21)	4.87 (3.45–6.83)	**7.15 (5.17–9.81)**
Schizophrenic	3.07 (2.83, 3.34)	2.85 (1.71–4.70)	3.84 (2.44–5.99)	**5.69 (4.05–7.92)**
Antisocial	3.16 (2.89, 3.44)	**10.95 (8.19–14.49)**	**6.90 (5.14–9.14)**	**11.92 (9.57–14.75)**
Histrionic	1.76 (1.61, 1.92)	2.09 (1.21–3.60)	2.92 (1.78–4.74)	**3.88 (2.62–5.72)**
Conduct disorder	1.05 (0.92, 1.20)	1.23 (0.56–2.68)	0.94 (0.46–1.89)	1.59 (0.91–2.77)
Substance Use Disorders				
Alcohol	27.52 (26.10, 28.98)	**63.41 (58.38–68.16)**	**60.49 (56.39–64.46)**	**72.65 (69.26–75.80)**
Nicotine	15.86 (15.00, 16.76)	**44.26 (39.93–48.69)**	**35.61 (31.79–39.62)**	**48.66 (44.65–52.69)**
Cannabis	7.45 (6.79, 7.96)	**23.65 (19.81–27.94)**	**18.59 (15.40–22.27)**	**23.66 (20.24–27.46)**
Amphetamine	1.69 (1.46, 1.95)	**5.28 (3.45–7.99)**	**5.74 (3.97–8.24)**	**7.09 (5.47–9.14)**
Opiates	1.16 (1.00, 1.36)	**3.23 (2.07–5.01)**	**4.05 (2.52–6.43)**	**6.73 (4.88–9.21)**
Sedative	0.89 (0.78, 1.02)	**1.92 (1.08–3.39)**	**3.08 (1.97–4.78)**	**4.72 (3.17–6.98)**
Tranquilizer	0.84 (0.72, 0.97)	1.59 (0.89–2.85)	**2.50 (1.50–4.15)**	**4.20 (2.75–6.37)**
Inhalant/Solvent	0.25 (0.19, 0.32)	0.33 (0.10–1.09)	**2.12 (1.23–3.63)**	**1.66 (0.91–3.01)**
Cocaine	2.44 (2.23, 2.67)	**6.18 (4.63–8.21)**	**6.44 (4.72–8.73)**	**9.99 (7.65–12.95)**
Hallucinogen	1.43, (1.25, 1.63)	**4.75 (3.27–6.84)**	**4.76 (3.15–7.15)**	**5.84 (4.32–7.86)**
Heroin	0.18 (0.14, 0.24)	0.18 (0.05–0.60)	**0.84 (0.32–2.18)**	**0.67 (0.30–1.47)**
Psychotic disorder	0.77 (0.67, 0.89)	1.07 (0.50–2.32)	1.36 (0.72–2.54)	1.14 (0.40–3.23)
Cigarettes Smoked				
Yes	41.74 (40.45, 43.03)	**70.12 (66.24, 73.72)**	**75.17 (71.66, 78.37)**	**74.37 (71.05, 77.44)**
No	58.26 (56.97, 59.54)	29.88 (26.28, 33.76)	24.83 (21.63, 28.34)	25.63 (22.56, 28.95)

Note: Boldface percentages are significantly different from those in the non-tobacco use group (p<0.05).

Table 3 presents associations between lifetime smokeless tobacco use and lifetime DSM-IV diagnoses of psychiatric disorders after controlling for sociodemographic covariates and cigarette smoking using a multinomial logit model. The adjusted odds of chewing tobacco in people with panic disorder and specific phobia were 1.53 and 1.41 times theodds in people without panic disorder and specific phobia, respectively. Alcohol use disorder was associated with 1.97, 2.01, and 2.99 times the odds of using snuff only, chewing tobacco only, and dual use of both tobacco products, respectively. Cannabis use disorder was associated with 1.44 times the odds of exclusive snuff use compared to non-cannabis use disorder. Opioid use disorder was associated with 1.81 times the odds of dual use of snuff and chewing tobacco. Inhalant and solvent use disorder was associated with 3.33 times the odds of exclusive chewing tobacco compared to non-inhalant/solvent use disorder.

**Table 3:** Adjusted odds ratios (OR) and 95% confidence intervals for lifetime psychiatric disorders while controlling for sociodemographic characteristics and quantity of cigarettes smoked.

	Snuff	Chewing tobacco	Snuff and chewing tobacco
	OR (95%CI)	OR (95%CI)	OR (95%CI)
Mood disorders			
Major depression	1.00 (0.77, 1.30)	0.78 (0.58, 1.05)	1.06 (0.80, 1.41)
Dysthymia	0.96 (0.54, 1.71)	1.42 (0.93, 2.18)	1.18 (0.74, 1.88)
Mania/hypomania	1.00 (0.68, 1.47)	1.09 (0.77, 1.55)	0.90 (0.66, 1.22)
Anxiety disorders			
Panic disorder	0.83 (0.45, 1.53)	**1.53 (1.01, 2.30)**	0.65 (0.37, 1.15)
Social phobia	1.19 (0.76, 1.86)	1.15 (0.72, 1.83)	0.72 (0.49, 1.05)
Specific phobia	1.03 (0.73, 1.45)	**1.41 (1.04, 1.92)**	0.87 (0.65, 1.17)
General Anxiety Disorder	0.78 (0.45, 1.34)	0.68 0.43, 1.09)	0.99 (0.56, 1.75)
Personality Disorder			
Avoidant	1.10 (0.57, 2.11)	1.00 (0.51, 1.98)	1.01 (0.54, 1.89)
Depressive	0.66 (0.15, 2.92)	0.78 (0.19, 3.10)	1.40 (0.49, 4.00)
Obsessive-Compulsive	1.11 (0.77, 1.61)	1.10 (0.81, 1.50)	1.29 (0.97, 1.71)
Paranoid	1.37 (0.84, 2.24)	0.94 (0.59, 1.48)	1.12 (0.75, 1.67)
Schizoid	0.58 (0.33, 1.02)	0.87 (0.52, 1.47)	1.18 (0.77, 1.81)
Antisocial	1.23 (0.86, 1.77)	0.75 (0.52, 1.06)	1.18 (0.86, 1.61)
Histrionic	0.61 (0.30, 1.22)	1.27 (0.74, 2.18)	1.11 (0.72, 1.72)
Conduct disorder	0.78 (0.35, 1.75)	0.65 (0.30, 1.40)	1.05 (0.59, 1.89)
Substance Use Disorders			
Alcohol	**1.97 (1.59, 2.45)**	**2.01 (1.65, 2.44)**	**2.99 (2.46, 3.62)**
Cannabis	**1.44 (1.09, 1.90)**	1.34 (0.99, 1.81)	1.12 (0.87, 1.46)
Amphetamine	1.41 (0.73, 2.71)	1.56 (0.89, 2.73)	1.29 (0.85, 1.98)
Opioid	1.08 (0.58, 2.02)	1.35 (0.65, 2.79)	**1.81 (1.09, 3.03)**
Sedative	0.83 (0.40, 1.72)	0.82 (0.41, 1.62)	1.41 (0.62, 2.11)
Tranquilizer	0.46 (0.21, 1.05)	0.55 (0.27, 1.12)	0.59 (0.29, 1.20)
Inhalant/Solvent	0.39 (0.12, 1.34)	**3.33 (1.58, 7.04)**	1.19 (0.49, 2.91)
Cocaine	0.76 (0.50, 1.18)	0.80 (0.46, 1.39)	1.10 (0.73, 1.67)
Hallucinogen	1.01 (0.61, 1.69)	1.06 (0.55, 2.04)	0.74 (0.45, 1.20)
Psychotic disorder	1.29 (0.52, 3.23)	1.19 (0.55, 2.58)	0.85 (0.20, 3.62)

Note: All odds ratios were adjusted for the demographic covariates, quantity of cigarette smoked and the rest psychiatric correlates presented in the table. Boldface odds ratios are significantly significant (p<0.05).

Table 4 shows multivariate analysis results by comparing exclusive snuff or chewing tobacco use anddual use with respect to psychiatric disorders after controlling for demographic covariates. Compared to the dual use of smokeless tobacco, the likelihood of exclusive snuff use wasreduced by 35% and 52%, respectively, for people with alcohol use disorder and schizoid personality disorder. Opioid use disorder was not associated with the greater odds of dual use of snuff and chewing tobacco versus exclusive use of either product. The odds of chewing tobacco were reduced by 42% and 33%, respectively, for people with antisocial personality disorder and alcohol use disorder. In contrast, panic disorder, specific phobia and inhalant or solvent use disorder were associated with greater odds of exclusive chewing tobacco compared to dual use.

**Table 4:** Associations between lifetime psychiatric disorders and exclusive smokeless tobacco use status compared to dual use of snuff and chewing tobacco while controlling for sociodemographic characteristics and quantity of cigarettes smoked.

	Snuff	Chewing tobacco
	OR (95%CI)	OR (95%CI)
Mood disorders		
Major depression	0.98 (0.70–1.38)	0.77 (0.53–1.13)
Dysthymia	0.72 (0.38–1.33)	1.13 (0.62–2.05)
Mania/hypomania	1.24 (0.77–1.99)	1.29 (0.82–2.02)
Anxiety disorders		
Panic disorder	1.13 (0.49–2.59)	**2.24 (1.09–4.64)**
Social phobia	1.57 (0.87–2.85)	1.73 (0.96–3.12)
Specific phobia	1.22 (0.76–1.95)	**1.71 (1.15–2.53)**
General Anxiety Disorder	0.79 (0.39–1.60)	0.61 (0.32–1.17)
Personality Disorder		
Avoidant	1.27 (0.51–3.15)	0.92 (0.35–2.40)
Dependent	0.45 (0.06–3.46)	0.55 (012–2.53)
Obsessive-Compulsive	0.85 (0.53–1.37)	0.91 (0.61–1.34)
Paranoid	1.20 (0.65–2.21)	0.84 (0.45–1.59)
Schizoid	**0.48 (0.25–0.94)**	0.67 (0.33–1.38)
Antisocial	1.06 (0.69–1.63)	**0.58 (0.38–0.90)**
Histrionic	0.57 (0.24–1.35)	1.36 (0.67–2.74)
Conduct disorder	0.69 (0.26–1.85)	0.69 (0.28–1.72)
Substance Use Disorders		
Alcohol	**0.65 (0.49–0.86)**	**0.67 (0.51–0.89)**
Quantity of cigarettes	1.01 (0.94–1.09)	1.01 (0.94–1.09)
Cannabis	1.23 (0.84–1.79)	1.18 (0.81–1.72)
Amphetamine	0.91 (0.46–1.82)	1.09 (0.60–1.99)
Opioid	0.50 (0.24–1.06)	0.66 (0.29–1.52)
Sedative	0.79 (0.27–2.38)	0.94 (0.34–2.56)
Tranquilizer	0.83 (0.26–2.63)	0.76 (0.29–2.00)
Inhalant/Solvent	0.38 (0.08–1.86)	**3.16 (1.11–9.03)**
Cocaine	0.66 (0.37–1.18)	0.73 (0.36–1.50)
Hallucinogen	1.65 (0.73–3.75)	1.60 (0.70–3.53)
Psychotic disorder	1.08 (0.30–3.93)	1.41 (0.46–4.30)

Note: All odds ratios were adjusted for the demographic covariates and the rest psychiatric correlates presented in the table. Boldface odds ratios are significantly significant (p<0.05).

## Discussions

We have yielded some new findings by including a broader range of psychiatric correlates, using a lifetime diagnosis and specifying subtypes of smokeless tobacco use. Unlike cigarette smoking, which is associated with a broad range of psychiatric disorders, lifetime smokeless tobacco use is associated with a subset of lifetime diagnoses of psychiatric disorders. The profile of lifetime psychiatric correlates is different depending on the type of smokeless tobacco used. For example, panic disorder and specific phobia were associated with a greater likelihood of exclusive chewing tobacco in our study, consistent with a positive association between smokeless tobacco use and specific phobia was reported among people with nicotine dependence previously [10]. A positive association between lifetime cannabis use disorder and lifetime exclusive snuff use was found in this study, inconsistent with the previous study which concluded that smokeless tobacco use was not associated with cannabis use disorder [11]. We demonstrated a strong positive association between inhalant or solvent use disorder and exclusive chewing tobacco, which had not been reported previously. Alcohol use disorder is associated with all types of smokeless tobacco use. Furthermore, compared to dual use of snuff and chewing tobacco, exclusive use of either type of smokeless tobacco product is uniquely associated with different lifetime diagnoses of psychiatric disorders.

The present study is different from the previous studies [10], [11] in several important ways. First, in the previous studies, smokeless tobacco use was treated as an independent variable and psychiatric disorders were dependent variables. In the present study, smokeless tobacco use was treated as a dependent variable and psychiatric disorders were independent variables. This analytical approach facilitates detection of whether each psychiatric disorder is independently associated with smokeless tobacco use. Second, unlike previous studies that combined snuff use and chewing tobacco together and focused on comparisons between smoked and smoked plus smokeless tobacco use, the present study exclusively focused on smokeless tobacco use. As such, we were able to examine the effect of specific types of smokeless tobacco use. Third, to maximize the sample size of smokeless tobacco use, lifetime smokeless tobacco users were included in the analysis. In contrast, Goodwin et al. [10] study included only past-year smokeless tobacco users. Agrawal and Lynskey's [11] study divided smokeless tobacco users into exclusive smokeless tobacco users and smoke plus smokeless tobacco use. As a result, the sample size of smokeless tobacco users is significantly reduced. In the present study, it was recognized that many smokeless tobacco users were also smokers. We controlled the smoking effects by including quantities of cigarettes smoked as a covariate. Finally, the relation of smokeless tobacco use with cannabis use and cannabis use disorder was the central focus in a previous study [11], while the present study examined a broad spectrum of psychiatric disorders in relation to each specific type of smokeless tobacco use.

Exclusive smokeless tobacco use has been linked to alcohol use disorder [11]. Our study extends the previous finding by demonstrating that alcohol use disorder is associated with all types of smokeless tobacco use. Moreover, the association between dual use and alcohol use disorder is much stronger than that between exclusive use of either type of smokeless tobacco and alcohol use disorder. This strong association cannot be explained by cigarette smoking, sociodemographics and other psychiatric disorders. The biological mechanism of the association between smokeless tobacco use and alcohol use disorder is not clear. Little research has elucidated the biological link between smokeless tobacco and alcohol dependence. Alcohol dependence and cigarette smoking are associated with low platelet monoamine oxidase (MAO) activity levels. Cigarette smoking leads to the binding of an inhibitor contained in cigarette smoke to platelet MAO-B at the catalytic site of MAO. However, a recent Swedish study compared the MAO activity of three groups of tobacco use among alcohol dependent subjects: non-tobacco users (n = 46), snuff users (n = 14), and cigarette users (n = 33) [24]. The MAO activity levels were significantly lower in cigarette smokers, but not in snuff users, compared to non-tobacco users. This finding suggests that snuff use may not have an inhibitory effect on MAO activities among alcohol dependence users. It is unknown if this phenomenon can be observed in people without alcohol dependence. Low aldehyde dehydrogenase (ALDH) activity is a marker of alcohol dependence. The mean whole blood ALDH activity of the smokers was found to be reduced significantly in smokers, but not in snuff users [25], even though cigarette smokers and snuff users had similar plasma nicotine and cotinine levels. The investigators speculated that the reduced blood ALDH activity in smokers was not caused by nicotine or any of its metabolites, but more likely, by components formed during the combustion of tobacco. In combination, these studies suggest that the biological mechanism underlying the association between cigarette use and alcohol use disorder cannot be automatically extrapolated to the relationship between smokeless tobacco use and alcohol use disorder. More research is needed to uncover the underlying mechanism.

The present study did not identify a significant relationship between possession of any mood disorder and smokeless tobacco use, consistent with a previous report in the USA[10], but inconsistent with a Finnish adolescent longitudinal study that found that early onset depressive disorders predicted two times the risk for smokeless tobacco use three years later [26]. These inconsistent findings could be attributable to study design (i.e. longitudinal vs. cross-sectional), difference in controlling for confounding effects, and different cultural factors that exist between Finland and USA.

The finding of an association between inhalant/solvent use disorder and exclusive chewing tobacco is quite novel. People who had a lifetime diagnosis of inhalant or solvent use disorder are three times more likely to chew tobacco than those without inhalant/solvent use disorder. Previous studies have shown that inhalant abuse is usually initiated during preadolescence [27] and chewing tobacco often begins in adulthood [28]. Therefore, people with a history of inhalant abuse may be at high risk for chewing tobacco at some point in their life. Our novel finding may inform the primary prevention effort for chewing tobacco use. Future research that uses a prospective design is needed to confirm our hypothesized pathway.

Dual use of snuff and chewing tobacco versus exclusive use of one of the two types of smokeless tobacco has not been investigated previously. The present study found that both panic disorder and specific phobia were associated with greater odds of exclusive chewing tobacco rather than exclusive snuff use and dual use of both smokeless tobacco products. These findings suggest that chewing tobacco is specifically linked to panic disorder and specific phobia. In addition, schizoid and antisocial personality disorder is less likely to be associated with exclusive use of snuff and chewing tobacco, respectively, than dual use. These results may inform secondary prevention of smokeless tobacco use. Depending on different types of smokeless tobacco used, one or two particular psychiatric comorbidities should be taken into account during the treatment.

The present study's results should be interpreted within the context of several limitations. Because present findings were derived from a cross-sectional study, causal relations between smokeless tobacco and psychiatric disorders cannot be determined. Lifetime diagnoses of psychiatric disorders and lifetime smokeless tobacco use used in this study may obscure some true relationships between smokeless tobacco use and psychiatric disorders. It is also possible that the observed associations are due to a common cause that was not measured in this study. Future studies employing prospective design are needed to unravel the mechanism that underlies the identified associations. The present study's findings were derived based on interviews with adults 18 years or older included in the NESARC dataset and the vast majority of smokeless users were men. Thus, results may not apply equally well to the adolescent population. Future research in that special population is needed to confirm current findings. Finally, we could not examine the subtypes of smokeless tobacco use in relation to heroin use disorder due to a small proportion of smokeless tobacco users who met the lifetime DSM-IV diagnostic criteria for heroin use disorder.

Despite those limitations, this is the first epidemiologic study of its kind to systematically examine the associations between each specific type of lifetime smokeless tobacco use and lifetime diagnoses of psychiatric disorders. As such, these findings provide new insights into the link of psychiatric disorders with snuff use and chewing tobacco. These links are understudied yet given the public health significance of smokeless tobacco product and their health consequences. Thus, elucidating the link between psychiatric and behavioral health factors is important. These results can guide treatment efforts on smokeless tobacco by targeting the treatment for alcohol use disorder among all smokeless tobacco users, the treatment for panic disorder, specific phobia, and inhalant use disorder among exclusive chewing tobacco users, as well as the treatment for cannabis use disorder among exclusive snuff users. It is helpful to realize that persons who use both snuff and chewing tobacco are different from exclusive users of snuff or chewing tobacco regarding their comorbid antisocial and schizoid personality disorders, respectively.
